# Budget impact model of Mydrane®, a new intracameral injectable used for intra-operative mydriasis, from a UK hospital perspective

**DOI:** 10.1186/s12886-018-0746-x

**Published:** 2018-04-19

**Authors:** Keith Davey, Bernard Chang, Christine Purslow, Emilie Clay, Anne-Lise Vataire

**Affiliations:** 1 Calderdale and Huddersfield NHS Foundation Trust, Huddersfield, UK; 2 Leeds Teaching Hospitals NHS Trust, Leeds, UK; 3 Thea Pharmaceuticals, Keele, UK; 4 Creativ-Ceutical, Paris, France

**Keywords:** Cataract surgery, Mydriasis, Mydriatics, Anaesthesia, Budget impact model

## Abstract

**Background:**

During cataract surgery, maintaining an adequate degree of mydriasis throughout the entire operation is critical to allow for visualisation of the capsulorhexis and the crystalline lens. Good anaesthesia is also essential for safe intraocular surgery.

Mydrane® is a new injectable intracameral solution containing two mydriatics (tropicamide 0.02% and phenylephrine 0.31%) and one anaesthetic (lidocaine 1%) that was developed as an alternative to the conventional topical pre-operative mydriatics used in cataract surgery. This study aimed to estimate the budget impact across a one year time frame using Mydrane® instead of topical dilating eye drops, for a UK hospital performing 3,000 cataract operations a year.

**Methods:**

A budget impact model (BIM) was developed to compare the economic outcomes associated with the use of Mydrane® versus topical drops (tropicamide 0.5% and phenylephrine 10%) in patients undergoing cataract surgery in a UK hospital. The outcomes of interest included costs and resource use (e.g. clinician time, mydriasis failures, operating room time, number of patients per vial of therapy etc.) associated with management of mydriasis in patients undergoing cataract surgery. All model inputs considered the UK hospital perspective without social or geographical variables. Deterministic sensitivity analyses were also performed to assess the model uncertainty.

**Results:**

Introduction of Mydrane® is associated with a cost saving of £6,251 over 3,000 cataract surgeries in one year. The acquisition costs of the Mydrane® (£18,000 by year vs. £3,330 for eye drops) were balanced by substantial reductions in mainly nurses’ costs and time, plus a smaller contribution from savings in surgeons’ costs (£20,511) and lower costs associated with auxiliary dilation (£410 due to avoidance of additional dilation methods). Results of the sensitivity analyses confirmed the robustness of the model to the variation of inputs. Except for the duration of one session of eye drop instillation and the cost of Mydrane®, Mydrane® achieved an incremental cost gain compared to tropicamide/phenylephrine eye drops.

**Conclusions:**

Despite a higher acquisition cost of Mydrane®, the budget impact of Mydrane® on hospital budgets is neutral. Mydrane® offers a promising alternative to traditional regimes using eye drops, allowing for a better patient flow and optimisation of the surgery schedule with neutral budget impact.

## Background

Cataract surgery is a very common procedure that is anticipated to increase in the coming years with the expected aging of the population [1, 2]. In 2014–2015, there were a total of 396,000 cataract surgeries performed in England [3]. Phacoemulsification is the most common procedure; it is minimally invasive, and mostly completed in an outpatient setting involving an expedited post-operative recovery [4].

During this procedure, induction of pupil mydriasis and maintenance of an adequate degree of mydriasis throughout the entire operation are critical for successful lens removal and replacement [5, 6]. Additionally, good anaesthesia is essential for the performance of safe intraocular surgery, creating a comfortable environment for the patient and the surgeon during surgery. Insufficient mydriasis or pupillary constriction due to surgical trauma can lead to several risks in the course of surgery, including incomplete cortex removal, posterior capsule rupture, vitreous loss and dislocation of lens material [7]. Insufficient mydriasis is also a source of discomfort for the surgeon as it makes instrument manoeuvring within the eye difficult. If mydriasis fails during the surgical process, surgeons might utilise rescue mydriatic therapies and procedures (e.g. ophthalmic injections and iris hooks) to re-dilate the pupils and maintain iris retraction and/or control iris floppiness [5, 6].

The standard combination of two topical mydriatic drugs (parasympatholytic and sympathomimetic, typically tropicamide or cyclopentolate, and phenylephrine) to achieve appropriate pre-operative mydriasis presents several disadvantages. From the nurse’s perspective, mydriatic eye drop application is time-consuming and stressful associated with a risk of mistakes, leading to delay of the surgery or risks of overdosing. From the patient’s perspective, mydriasis with drops involves a long waiting time, which may increase his/her anxiety and stress before cataract surgery. Moreover, the unpredictable time to mydriasis can make it difficult to control the flow of patients ready to be operated and to optimize the surgical schedule. The use of rescue mydriatics by the surgeon occurs in 15 to 18% of the operations and may lead to delays in theatre [8]. Specifically, the time taken to achieve maximal mydriasis can be much longer than the surgical procedure itself.

Mydrane® was developed as an alternative to the conventional topical pre-operative mydriatics for cataract surgery. It is an intracameral (IC) injectable solution containing a combination of two mydriatics (tropicamide 0.02% and phenylephrine 0.31%) and one anaesthetic (lidocaine 1%). It is injected intracamerally under topical anaesthesia by the surgeon at the beginning of the surgery through the side or primary port. Due to the specific, localised therapy, Mydrane® was shown to achieve fast onset of mydriasis and low systemic absorption [7]. Additionally, Mydrane’s administration route alleviates the limitations of the topical mydriatics [9].

Since the number of cataract procedures is increasing due to the combined effects of population ageing and higher life expectancy, innovative treatments are essential to improve cataract surgery efficiency with the aim to regulate patient’s flow with moderate health care expenditure. For this purpose, we developed an economic model to estimate the budget impact for a UK hospital performing cataract surgeries of Mydrane® for injection use instead of topical dilating eye drops over a one year time-frame.

## Methods

A budget impact model (BIM) was developed using Microsoft Excel 2010 (Microsoft Corp., Redmond, WA, USA) to compare the economic outcomes associated with utilisation of Mydrane® versus topical eye drops (tropicamide 0.5% and phenylephrine 10%) in adult patients undergoing phacoemulsification cataract surgery in a UK hospital. The outcomes of interest include cost and resource use (e.g. clinician time, mydriasis failures, operating room time, number of patients per vial of therapy etc.) associated with management of mydriasis in patients undergoing cataract surgery. All model inputs considered the UK hospital perspective without social or geographical variables.

### Model settings

The budget impact model predicted health care costs of one year for cohorts of adult patients with cataract to be operated by phacoemulsification, in a given hospital, in the UK in two situations: the use of Mydrane® (intervention group) and the use of topical eye drops (tropicamide (0.5%) and phenylephrine (10%)) for mydriasis (reference group). It was assumed that 3,000 patients a year could be operated for cataract.

**Fig. 1:**
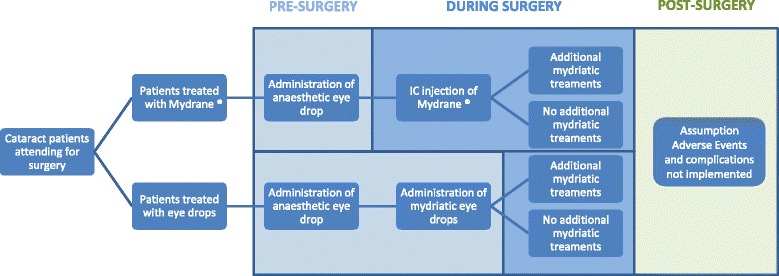
Budget Impact Model Structure

A decision tree was chosen to represent the patient’s pathway (Fig. 1). The model was divided into three time phases: pre-surgery (pre-operative), surgery (intra-operative) and post-surgery (post-operative). At each state of the decision tree, resource use and costs were calculated and summed together to obtain total costs with respect to each treatment strategy. 1) The pre-surgery period included all administered interventions and outcomes achieved between the time of the patient’s arrival at the hospital and the time of the ophthalmologist initial surgical methods. The nurse is typically the key person during the pre-operative phase of the cataract surgery procedure. She/he takes care of the patient before his/her arrival at the operating room and undertakes most of the care before the surgical procedure, including checking the condition of the eye and dilating the pupil with mydriatic eye drops. Anaesthesia costs were not taken into account as it is similar for the two strategies. 2) The surgery period was defined as the complete time of cataract surgery and included all methods, resources, and outcomes achieved through completion of the procedure. The surgical procedure can be divided into 2 main phases, a first phase of preparation involving the ocular surface and a second phase involving tissue manipulations inside the anterior chamber. 3) Finally, the post-surgery period was defined as the time period following all surgical activities by the ophthalmologist, which may include patients receiving medication for the prevention of post-operative infections and alleviation of ocular inflammation and pain [10, 11].

### Clinical treatment protocol used in the model

Patient treatment groups differed in frequency and timing of drugs administered during the pre-surgery and surgery phases. For cataract patients treated with standard care (topical eye drops), the administration schedule included instillation of one drop of tropicamide 0.5% and one drop of phenylephrine 10% instilled by nurses in three instances; at 30, 20, and 10 min prior to surgery. In the Mydrane® group, the surgeon injected Mydrane® 200 μL IC at the beginning of surgery. Both patient groups also received topical anaesthetics 5 min and 1 min prior to surgery. Based on the phase III LT2380-PIII-05/10 clinical trial data [9], these treatment strategies were well tolerated and did not differ in their short-term adverse events profiles, hence these variables were not incorporated in the model.

Following appropriate administration of mydriatic and local anaesthetic, the surgeon proceeded with phacoemulsification. If adequate dilation was not achieved, additional therapy or mechanical methods were used. When such steps were taken, the model took account of this (e.g. increased waiting time during surgical procedures).

### Model inputs and outcomes

All model inputs (with associated sources) are presented in Table 1. Pre-surgery inputs included proportion of mydriasis failures, quantity of eye drop instillations, number of patients per vial of therapy, and nursing time costs. During the surgery phase, many of the pre-surgery inputs carried over, with additional inputs for operating room occupancy time, cost of treatments, time for mydriasis failure, surgeon cost and procedure time. The number of annual procedures was calculated by dividing the total surgeon working time gained by the time needed for a cataract surgery. Additionally, hospital revenue was calculated based on the number of operations performed by each surgeon. The structure of the model and its conformity with UK clinical practice was validated by the co-authors with clinical expertise.

**Table 1:** Model inputs

Variable		Base case	Low value	High value	Source
Pre-surgery					
Mydrane®: Percentage of mydriasis failure during surgery needing additional mydriatic treatments					
Mydrane®		1.1%	0.2%	3.2%	Phase III clinical trial results, Confidence Interval 95% [9]
Eye drops		5.3%	3.0%	8.7%	
Number of eye drop instillation sessions by nurses		3	2	4	Assumption
Duration of one session of eye drop (min)		3	1.5	5	Assumption, Mydriasert report, Office for National Statistics [15]
Number of patients per vial of eye drops		1	1	5	Assumption, Mydriasert report, Office for National Statistics [15]
Patient waiting time before surgery (min)					
Mydrane®		8.70	8.19	9.21	Phase III clinical trial results, Confidence Interval 95% [9]
Eye drops		37.90	36.73	39.07	
Nurse cost/h in £		43.12	35.76	59.95	PSSRU 2012–2013, Lower: nurse day ward without qualification costs, Higher: nurse team manager [16]
During surgery					
Mydrane®: Surgeon working time (no mydriasis failure) (min)		12.03	11.51	12.52	Phase III clinical trial results [9], Phase II clinical trial results, Confidence Interval 95%
Eye drops: Surgeon working time (no mydriasis failure) (min)		11.34	10.64	12.04	Phase III clinical trial results, Confidence Interval 95% [9]
Mydrane®: Operation room occupancy time (min)		12.03	11.51	12.52	Phase III clinical trial results [9], Phase II clinical trial results, Confidence Interval 95%
Eye drops: Operation room occupancy time (min)		11.34	10.64	12.04	Phase III clinical trial results [9], Confidence Interval 95%
Mydrane®: Additional time needed if mydriasis failure (min)		10.00	2.50	7.50	UK, 2013, Molecule used for additional mydriasis, European Observatory for Cataract Surgery 2014 [7]
Eye drops: Additional time needed if mydriasis failure (min)		10.00	2.50	7.50	UK, 2013, Molecule used for additional mydriasis, European Observatory for Cataract Surgery 2014 [7]
Mydrane®: Surgeon time loss between operations caused by mydriasis failure (min)		10.00	5.00	20.00	Assumption
Eye drops: Surgeon time loss between operations caused by mydriasis failure (min)		10.00	5.00	20.00	Assumption
Additional mydriatic treatments distribution (more than 1 possible)	Adrenaline	4%	39%	4%	UK, 2013, Molecule used for additional mydriasis, European Observatory for Cataract Surgery 2014 [7]
	Phenylephrine	93%	57%	93%	
	Tropicamide	14%	0%	14%	
	Mechanical tools	4%	5%	4%	
	Cyclopentolate	32%	1%	32%	
	Others	0%	0%	0%	
Cost Mydrane® in £		6.00	3.00	9.00	Assumption
Cost Adrenaline in £		4.95	0.39	8.31	British National Formulary 2015 [17]
Cost Phenylephrine in £		0.57	0.57	0.57	British National Formulary 2015 [17]
Cost Tropicamide in £		0.54	0.50	1.60	British National Formulary 2015 [17]
Cost Mechanical tools in £		56.80	28.40	85.20	Mydriasert pupillary dilation for cataract surgery.
Costs Cyclopentolate in £		0.56	0.50	12.96	British National Formulary 2014
Surgeon cost/h in £		147.26	105.18	189.33	Lower: consultant surgery without costs including qualifications, Upper: assumption

### Costs of treatment strategies

The reference treatment group was composed of patients treated with one drop of tropicamide 0.5% and one drop of phenylephrine 10% instilled by nurses in three instances at 30, 20 and 10 min prior to surgery. Comprehensive clinical information, resource use and unit cost are presented in Table 3. Costs were expressed in British pounds (£, January 2015).

### Sensitivity analysis

Deterministic sensitivity analyses were performed to assess the model uncertainty. All input parameters are varied one by one according to bounds defining the possible range of variation of the considered parameter. These analyses ran the model while simultaneously varying one specific variable and holding all others constant. This process tests the model assumptions and parameter estimates validity, given their uncertainty. Parameters of the model were varied using data from alternative publications or confidence intervals obtained from clinical trials. In case of no available relevant information on uncertainty, the base case value was between 50 to 150%. The list of inputs used in the deterministic sensitivity analysis is described in Table 1. All deterministic and sensitivity analyses were then compared between groups based on incremental costs calculated according to the various inputs.

## Results

### Resource outcomes

**Fig. 2:**
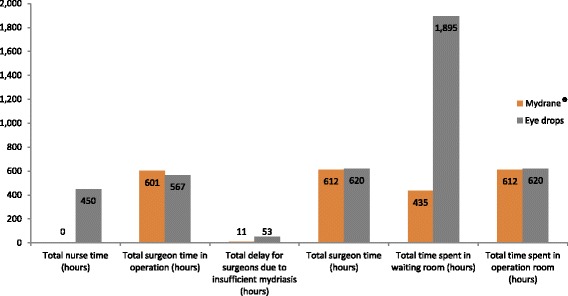
Base case results – Resource use comparison (yearly basis)

Overall, Mydrane® was found to be a beneficial treatment reducing costs from the hospital, surgeon, and nurse perspectives (Table 2 and Fig. 2). With respect to hospital outcomes, Mydrane® decreased the time spent in the waiting rooms. Specifically, during the pre-surgery phase, patients’ total time in the waiting room was lower compared to the reference group (435 vs. 1895 h, respectively; incremental difference 1460 h).

**Table 2:** Base case results - Resource use

	Mydrane®	Eye drops	Difference Mydrane® - Eye drop
Occupation of rooms			
Total time spent in waiting room (hours)	435.00	1895.00	-1460.00
Total time spent in operation room (hours)	612.25	619.75	−7.50
Surgeon time			
Number of unexpected delays due to insufficient mydriasis during surgery	33.00	159.00	−126.00
Total surgeon time in operation (hours)	601.25	566.75	34.50
Total delay for surgeons due to insufficient mydriasis (hours)	11.00	53.00	−42.00
Total surgeon time (hours)	612.25	619.75	−7.50
Nurse time			
Total nurse time (hours)	0.00	450.00	−450.00

The number of unexpected delays caused by mydriasis failure was significantly reduced between treatment groups [33 (Mydrane®) vs. 159 (tropicamide/phenylephrine) delay cases; difference − 126]. Throughout the year, surgeon time delay was reduced of 42 h, decreasing from 53 h for the reference group to 11 h for the Mydrane® group. However, patients in the Mydrane® group experienced more “working time” in surgery (601 h vs. 567 h; difference 35 h); subsequently, considering the pre-operative mydriasis time savings, the total procedure time was lower for the Mydrane® group (7 h saved over one year). Lastly, in terms of nursing time, Mydrane® patients did not need additional topical eye drop instillations to achieve mydriasis and this time was freed up (450 h over one year).

### Economic outcomes

**Fig. 3:**
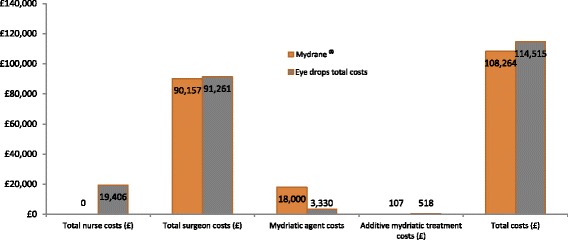
Base case results – Costs comparison (yearly basis)

In the two treatment groups, the predicted total costs over one year were £108,264 (Mydrane®) and £114,515 (tropicamide/phenylephrine) (Table 3 and Fig. 3). We assumed that one vial of each eye drop per patient was used in line with good clinical practice and product licence. Therefore, the Mydrane® strategy realised an annual cost saving of £6,251 and supported budget impact savings from a hospital perspective. Mydrane® also decreased hospital staff costs compared to traditional topical tropicamide/phenylephrine drops. Specifically, significant cost savings were obtained in nursing time to instil drops in the pre-operative period. Over one year, cost savings amounted to £19,406.

**Table 3:** Base case results – Costs

	Mydrane®	Eye drops	Difference Mydrane® - Eye drop
Total nurse costs (£)	0	19,406	−19,406
Total surgeon costs (£)	90,157	91,261	− 1104
Total work-related costs (£)	90,157	110,668	−20,511
Mydrane® costs (£)	18,000	0	18,000
Eye drop costs (£)	0	3330	−3330
Additive mydriatic treatment costs (£)	107	518	−410
Total treatment-related costs (£)	18,107	3848	14,260
Total costs (£)	108,264	114,515	−6251
Costs per patient (£)	36.09	38.17	−2.08

The predicted annual work-related costs were similar in the Mydrane® and the topical eye drops arms (£90,157 vs. £91,261, respectively). The Mydrane® strategy gave the surgeon additional working time. Overall, using Mydrane® provided a total net cost saving as a consequence of the reduction of cases with insufficient mydriasis. Furthermore, compared to the Mydrane® group, the topical eye drops group had higher predicted treatment costs due to additional mydriatic therapies required for patients who initially failed mydriasis (£518 vs. £107, respectively; Table 3). Overall, the predicted treatment related costs were lower among patients in topical eye drops group compared to the Mydrane® group (£3848 vs. £18,107, respectively).

Surgeons experienced reduced working time for patients treated with Mydrane®; thus, allowing for more efficient clinical throughput and providing opportunities for resource optimisation. More specifically, considering the increased time to perform additional operations, the model indicated that changing to Mydrane® could potentially free up sufficient time to allow approximately 36.81 more cataract procedures per year, bringing in an additional £27,412 in revenue (Table 4).

**Table 4:** Results: Benefits for the hospital

	Consequences of Mydrane® introduction
Total number of operations gained	36.81
Total additional revenue (£)	27,412
Total expected net benefit (£)	11,733

### Sensitivity analysis

**Fig. 4:**
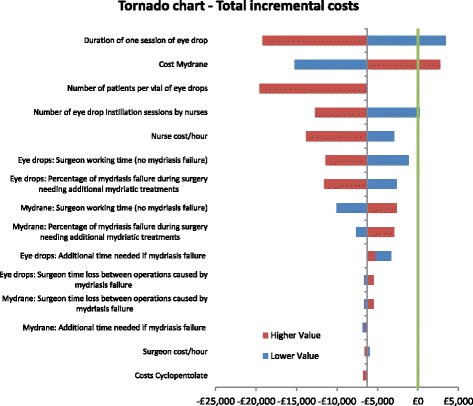
Deterministic sensitivity analysis results. *Green line: No economic impact (null incremental cost). Grey line: Base case result*

The tornado diagram presented in Fig. 4 illustrates the results of the deterministic sensitivity analyses. Results of the sensitivity analyses confirmed the robustness of the model to the variation of inputs. Except for the duration of one session of eye drop instillation and the cost of Mydrane®, Mydrane® achieved an incremental cost gain compared to topical eye drops. The model results were most sensitive to the following parameters: duration of one session of eye drop instillation, cost of Mydrane®, number of eye drop instillations sessions by nurses, and nursing costs.

## Discussion

A budget impact model was developed for the introduction of Mydrane® in the UK over a 1-year time horizon. The model showed that the introduction of Mydrane® is associated with cost savings of £6398 by year for the hospital. The acquisition costs of Mydrane® compared to topical eye drops (£18,000/year vs. £3,330/year, respectively) were balanced by substantial reductions in nurses and surgeons costs (£20,658) and lower costs associated with distant recurrence (£410 due to additive mydriatic treatment avoided).

The BIM calculated similar costs between each treatment strategy and demonstrated the economic benefits of Mydrane® as an efficient treatment for mydriasis in cataract procedures. By producing a stable and fast mydriasis, it leads to less unexpected events, reduced lost time, freed up nursing time, optimised theatre time, and potentially reduces costs. Specifically, the time savings were achieved by decreasing the number of topical drop instillations and reduced mydriasis failure. The saved time potentially allows increased throughput with associated increased revenue. Our findings show that using Mydrane® instead of topical eye drops for cataract surgery patients should produce a net benefit for the hospital.

Additionally, Mydrane® treatment potentially provides a variety of indirect benefits to an adopting hospital. Amidst an aging population and capacity challenges, many patients face long waiting times for operations [12]. The savings achieved with respect to health care professionals’ time and prospective increase in patients treated per day, positions Mydrane® to positively impact the waiting time before surgery. Secondly, patients in the pre-operative area may experience deteriorated vision or increased anxiety as eye drops are applied; this may be alleviated by using an injectable treatment. Thirdly, patients treated with Mydrane® may be more likely to recover at a faster rate due to the lower risk of mydriatic failures and surgery complications. Overall, Mydrane® strategy offers reduced cost, improved health outcomes and minimised time losses from the institution, clinician, and patient’s perspectives.

While every effort was made to obtain key model inputs from the best available evidence, assumptions about some parameters were necessary for the budget impact estimation and may impact the model output. Therefore, limitations of this study need to be acknowledged. The model included a simple design to reflect the available data and transparency of calculations, but did not include all the specific processes of an ophthalmic surgery unit. The number of operations (3,000) is typical of an average ophthalmology unit in a UK city, and it is assumed that human resources and patient flow are already optimised for the throughput of the surgical unit, but it is possible that savings due to nurse time might be further influenced by local organisation. A literature search was conducted to identify previously published models for cataract surgery. In 2001, Lundström described a model comparing performance of different cataract surgery management in an ophthalmology department [8]. An index approach was modelled to measure cataract surgery outcome. Moreover, one previously microsimulation model included a detailed depiction of the cataract procedure process [13] but extended beyond the framework in Mydrane® clinical trial and could not be used in this model. Also, the number of procedures gained due to time saving and financial benefits for the hospital is not part of the BIM. However, for the purposes of this analysis, the methodology deviates from classic BIM structure and guidelines. Additionally, hospital room costs were not included in the BIM due to lack of official data.

The structure and primary components of this BIM are derived from clinical trials data [9]. Two deviations from the clinical trial protocol versus daily practice have to be considered in the model. In the Mydrane® group, according to the trial protocol, if mydriasis was not achieved following one injection, a second (lower dose) injection was indicated before rescue therapy. However, to appropriately represent clinical practice, Mydrane® patients received only one injection in the BIM. The percentage of mydriasis failure in the model was established taking into account data from patients that received one or two injections (causing an underestimation of the percentage of mydriasis failure), while the duration of operations used in the model corresponded to patients who had only one injection. Furthermore, the trial protocol required the surgeon to wait 1.5 min to proceed with operation, even if mydriasis was obtained immediately, although this wait is not standard in clinical practice. The Phase II LT2380-PII-11/07 trial showed that 95% of the pupil dilation (6.9 mm before viscoelastic injection) was reached in less than 30 s following Mydrane® administration [14]. Thus, following Mydrane® injection, more accurate mydriasis time estimation (29 s) was applied from a phase II clinical trial study and substituted in the BIM.

The sensitivity analyses indicated that the BIM was sensitive to several nurse-specific variables (e.g. duration of eye drops applications, number of eye drops instillations); both values were obtained after consultation with experts. Also, the patient’s (and any accompanying carer’s) perspective was not considered; because Mydrane® reduces waiting time for patients, the group would waste less time arriving early for drops to be applied and being monitored during that time.

## Conclusions

Despite a higher acquisition cost of Mydrane®, the economic impact is associated with benefits. Mydrane® represents a promising alternative to eye drops, permitting improved patient flow and optimization of the surgical schedule.

## Abbreviations

BIM: Budget impact model
IC: Intracameral
min: minutes
UK: United Kingdom
